# Preeclampsia Is Associated with Increased Central Aortic Pressure, Elastic Arteries Stiffness and Wave Reflections, and Resting and Recruitable Endothelial Dysfunction

**DOI:** 10.1155/2015/720683

**Published:** 2015-07-30

**Authors:** Juan Torrado, Ignacio Farro, Yanina Zócalo, Federico Farro, Claudio Sosa, Santiago Scasso, Justo Alonso, Daniel Bia

**Affiliations:** ^1^Centro Universitario de Investigación, Innovación y Diagnóstico Arterial (CUiiDARTE), Physiology Department, Faculty of Medicine, Republic University, 2125 General Flores, 11800 Montevideo, Uruguay; ^2^Department of Obstetrics and Gynecology “C”, Pereira Rossell Hospital, Faculty of Medicine, Republic University, 1550 Bulevar Artigas, 11600 Montevideo, Uruguay

## Abstract

*Introduction*. An altered endothelial function (EF) could be associated with preeclampsia (PE). However, more specific and complementary analyses are required to confirm this topic. Flow-mediated dilation (FMD), low-flow-mediated constriction (L-FMC), and hyperemic-related changes in carotid-radial pulse wave velocity (PWVcr) offer complementary information about “recruitability” of EF. *Objectives*. To evaluate, in healthy and hypertensive pregnant women (with and without PE), central arterial parameters in conjunction with “basal and recruitable” EF. *Methods*. Nonhypertensive (HP) and hypertensive pregnant women (gestational hypertension, GH; preeclampsia, PE) were included. Aortic blood pressure (BP), wave reflection parameters (AIx@75), aortic pulse wave velocity (PWVcf) and PWVcr, and brachial and common carotid stiffness and intima-media thickness were measured. Brachial FMD and L-FMC and hyperemic-related change in PWVcr were measured. *Results*. Aortic BP and AIx@75 were elevated in PE. PE showed stiffer elastic but not muscular arteries. After cuff deflation, PWVcr decreased in HP, while GH showed a blunted PWVcr response and PE showed a tendency to increase. Maximal FMD and L-FMC were observed in HP followed by GH; PE did not reach significant arterial constriction. *Conclusion*. Aortic BP and wave reflections as well as elastic arteries stiffness are increased in PE. PE showed both “resting and recruitable” endothelial dysfunctions.

## 1. Introduction

Preeclampsia/eclampsia (PE) syndrome, defined as new onset and persistent hypertension after 20 weeks of gestation in association with significant proteinuria [1], is a major cause of maternal-fetal morbidity and mortality worldwide. The pathophysiology of PE remains incompletely understood, and anticipation and appropriate management of this disorder are frequently insufficient [2]. The prevalent pathogenic theory of PE includes the manifestation of two characteristic and sequential processes considered to be of paramount importance. The first corresponds to an insufficient placentation, which drives an increase in the resistance of the uteroplacental circulation, and the second involves the maternal reaction through the activation of an inappropriate inflammatory response with a (proposed) globally impaired endothelial function (EF) [3]. Structural and functional alterations in large arteries have also been reported accompanying PE syndrome [4, 5].

Impaired EF and arterial damage could occur for a certain time before significant proteinuria and clinical manifestations of PE become apparent [3, 6]. Thus, the possibility of identifying early subclinical endothelial dysfunction, as well as structural and/or functional arterial alterations during pregnancy, could be of value in recognizing and classifying the different hypertensive disorders of pregnancy. Hopefully, this will have a positive impact on the understanding of this syndrome, as well as on the appropriate and early management of these patients.

Celermajer et al.'s technique, commonly known as* flow-mediated dilation* (FMD), utilizes the vascular (or vaso-) reactivity test (VRT) and has become the most popular method to assess EF [7]. The VRT consists of positioning a pneumatic cuff around the upper arm and provoking an arterial occlusion for five minutes (transient ischemia, TI). This maneuver elicits an increase in blood flow in the brachial artery once the cuff is deflated (i.e., reactive hyperemia, RH), which subsequently stimulates endothelium to release nitric oxide (NO). Finally, locally produced NO results in a dilation of the brachial artery (assessed by B-Mode ultrasound) [7] and a reduction of arterial stiffness (changes in pulse wave velocity (PWV) assessed by mechanotransducers [8]). The magnitude of the arterial dilation is used as an indicator of EF, and healthy pregnant women show an enhanced vascular response evaluated by this method compared with healthy nonpregnant women [9, 10]. Whereas FMD provides information about the “recruitability” of EF (i.e., its responsiveness to a specific stimulus), it does not provide information concerning basal/tonic EF (i.e., release of endothelial autacoids before FMD measures are initiated) [11]. In this context, Gori et al. described a novel index for assessing the response of the artery to low flow, which utilizes data obtained from the cuff occlusion period of an FMD test [12]. Synonymous to FMD, the vasoconstriction observed under conditions of reduced flow has been named low-flow-mediated vasoconstriction (L-FMC) [12]. Inclusion of L-FMC data to traditional measurement of FMD could provide additional and/or complementary information, which, they propose, may improve the detection of patients with cardiovascular disease and profile the vascular response to exercise among healthy volunteers [13]. Whether the integration of L-FMC into traditional FMD studies will provide additional/complementary information among patients with hypertensive disorders in pregnancy is unknown.

In addition, changes in arterial stiffness assessed by means of carotid-to-radial pulse wave velocity (PWVcr) due to the same test (VRT) have been proposed as an alternative tool for the evaluation of EF [8, 14]. PWV, in particular carotid-to-femoral PWV, is recognized as the “gold standard” parameter for the evaluation of regional aortic stiffness having a wide biomedical application [15]. A reduction in PWVcr values (i.e., upper limb region) in response to VRT has been evidenced in healthy young adults, whereas a blunted reduction has been reported in pathophysiological circumstances such as hypertension [16] and congestive heart failure [14]. However, the impaired EF (which could follow hypertensive disorders of pregnancy) can be assessed by using PWVcr changes and if it provides additional or complementary information to those of brachial diameter assessment has not been studied yet.

In this context, the aims of this work were as follows: firstly, to determine noninvasive central and peripheral arterial parameters in a group of healthy and hypertensive pregnant women, through the use of validated techniques and parameters; secondly, to determine and analyze “basal and recruitable” EF through the measurement of FMD, L-FMC, and PWVcr changes.

## 2. Methods

### 2.1. Subjects

This was a cross-sectional study involving 26 pregnant women. The normotensive subjects (healthy pregnant women, HP; *n* = 10) were recruited from the routine antenatal clinic. They were all healthy and without family history of premature heart disease. All women had uncomplicated pregnancies before and during the study. Women with preeclampsia (PE; *n* = 8) and with gestational hypertension (GH; *n* = 8) were recruited from the antenatal hospital ward, where they were admitted due to mild hypertension (140/90 to 149/109 mmHg).

The definitions used followed the classification of the gestational hypertensive disorders, as recommended by the report of the National Collaborating Centre for Women's and Children's Health, hypertension in pregnancy, of the National Institute for Health and Clinical Excellence [1]. Under this classification, PE was defined as BP greater than 140/90 mmHg on two consecutive occasions more than 4 h apart, in combination with significant proteinuria (>300 mg total protein in a 24 h urine collection) developing after 20 weeks of gestation in previously normotensive women. The same hemodynamic conditions without significant proteinuria defined gestational hypertension.

All PE included in the study were* mild* in terms of the severity of the syndrome. None of them received any vasoactive drugs. Participants were asked to abstain from physical activity and vitamin supplementation for at least 4 hours prior to the examination. Baseline demographic data were obtained by an obstetrician during a clinical interview and laboratory samples were extracted prior to the examination. The study protocol was approved by the Ethics Research Committee of the School of Medicine (Republic University, Uruguay) and all participants gave written informed consent.

### 2.2. Baseline Noninvasive Arterial Evaluation

After recompilation of clinical and laboratory data, subjects were instructed to lie in a left lateral position (to avoid vena cava compression by the uterus) in a temperature-controlled (21°–23°C) room, for at least 15 minutes, in order to establish stable hemodynamic conditions. Heart rate (HR) and right brachial (peripheral) systolic and diastolic blood pressure (pSBP and pDBP, resp.) were measured using an oscillometric device (Omron HEM-433INT Oscillometric System; Omron Healthcare Inc., Illinois, USA) at 2-minute intervals during the whole procedure. Mean blood pressure (MBP) was derived from the standard equation usually employed at the peripheral level: MBP = pDBP + 1/3(pSBP − pDBP).

#### 2.2.1. Carotid-to-Femoral Pulse Wave Velocity and Pulse Wave Analysis

**Figure 1:**
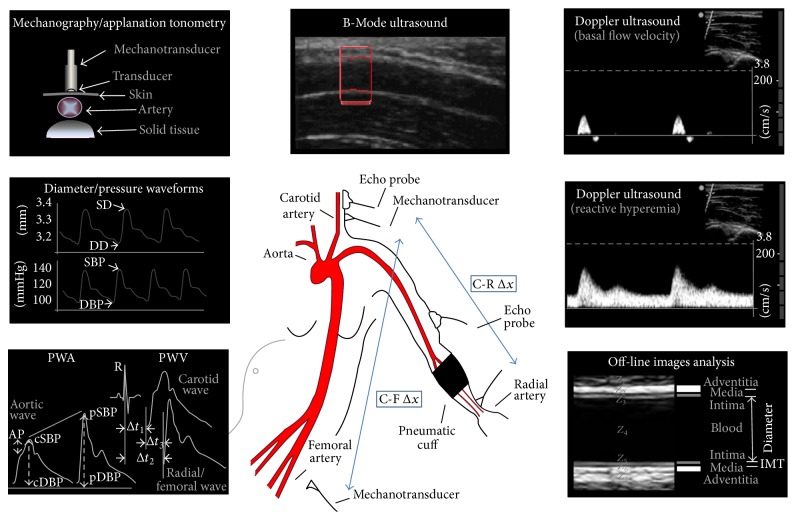
Schema of the instrumental approach employed to assess noninvasive arterial structure and function in basal and post-VRT conditions. Employed techniques are as follows: carotid-to-femoral and carotid-to-radial PWV (applanation tonometry and mechanography), PWA (applanation tonometry), arterial diameters and CIMT (B-Mode ultrasound), and blood flow velocities (Doppler ultrasound). PWA: pulse wave analysis; PWV: pulse wave velocity; AP: augmentation pressure; Δ*t*_1_, Δ*t*_2_: time delay between R wave from ECG and carotid foot wave and femoral/radial foot wave, respectively; Δ*t*_3_: time delay between carotid foot wave and femoral/radial foot wave; cSBP/pSBP: central/peripheral systolic blood pressure; cDBP/pDBP: central/peripheral diastolic blood pressure; DD: diastolic diameter; SD: systolic diameter; CIMT: carotid intima-media thickness; *Z*: acoustic impedance; C-F and C-R Δ*x*: carotid-to-femoral and carotid-to-radial distance, respectively.

The carotid-to-femoral pulse wave velocity (PWVcf) was measured to analyze aortic regional stiffness. To this end, carotid and femoral artery waveforms were consecutively obtained with a high-fidelity applanation tonometer from the carotid and femoral regions simultaneously with continuous ECG monitoring (SphygmoCor 7.01, AtCor Medical, Sydney, Australia) (Figure 1) [15]. Then, carotid-femoral propagation time (Δ*t*_3_) was determined by subtracting the time delay between the peak of R wave of the ECG recording to femoral foot of the pressure waveform (Δ*t*_2_) of the corresponding cardiac cycle and the time delay between the peaks of R wave to carotid foot of the pressure waveform (Δ*t*_1_) [15]. The algorithm utilized to detect the so-called “foot of the wave” was the* intersecting tangents*. Straight distance between the recording sites (carotid-to-femoral distance (C-F Δ*x*)) was then carefully measured using tape on the body surface to reduce the influence of altered body contour in pregnancy. Finally, PWVcf was automatically calculated as the quotient between C-F Δ*x* and Δ*t*_3_ (Figure 1) [15]. The reported value of PWVcf for a subject was always the average of at least eight consecutive beats.

Pulse wave analysis (PWA) was used to assess central hemodynamics as well as systemic arterial stiffness and wave reflections [15]. For this purpose, mean radial artery waveform was obtained (through the acquisition of many cycles) with the applanation tonometer from the wrist, and a corresponding mean ascending aortic pressure waveform was generated with a validated generalized transfer function using the same mentioned customized software (SphygmoCor 7.01, AtCor Medical, Sydney, Australia). The radial pulse waveform was then calibrated using the diastolic and mean arterial pressure obtained at the brachial artery [15]. Central systolic, diastolic, and pulse blood pressure (cSBP, cDBP, and cPP, resp.), heart rate (HR) corrected central augmentation index (AP/cPP × 100[%] heart rate adjusted to a HR of 75 bpm; AIx@75), and amplification ratio (pPP/cPP) were determined with the integrated software.

#### 2.2.2. Carotid Artery Studies

Ultrasound assessment of carotid arteries was based on the techniques and recommendations described in international consensus [17]. High-resolution B-Mode ultrasound images of both (right and left) common carotid arteries (CCAs) were obtained using a 10 MHz linear-array transducer connected to a portable ultrasound system (SonoSite, MicroMaxx, SonoSite Inc., 21919 30th Drive SE, Bothell, WA 98021, USA). Measurements (still images and video clips/cine loops) were digitally stored for off-line analysis (Figure 1). Near and far walls were analyzed and images were obtained from anterior, lateral, and posterior angles. At first, a carotid plaque screening was performed, for which the definition used was a focal structure that encroaches into the arterial lumen of at least 0.5 mm or 50% of the surrounding intima-media thickness or demonstrated a thickness of greater than or equal to 1.5 mm [17]. Then, longitudinal views of the CCAs were acquired and a video (cine-loop) of at least 10 seconds was recorded and stored. The CIMT and beat-to-beat diameter waveforms were obtained and analyzed off-line using a step-by-step border detection algorithm (based on changes in acoustic impedance (*Z*)), applied to each digitized image (Hemodyn-4M software, Buenos Aires, Argentina). A region of 1.0 cm proximal to the carotid bulb was identified, and the far wall CIMT was determined as the distance between the lumen-intima and the media-adventitia interfaces (Figure 1). The software performs multiple automated or semiautomated measurements along the centimeter and averages them, increasing the accuracy of the measures.

The instantaneous diameter (from the leading edge of the near wall intima-media interface to the intima-media interface of the far wall) waveform was obtained during pulsation in order to obtain diastolic and systolic arterial diameter. Then, complementary biomechanical parameters such as Peterson's elastic modulus (*E*_*P*_) and beta stiffness-index (*β*) were calculated relating these measures with central blood pressure as follows:

(1) $$\begin{array}{c} E_{P}=\frac{\left(\text{cSBP}-\text{cDBP}\right)}{\left(\text{SD}-\text{DD}\right)/\text{DD}},\\ \beta =\frac{\text{Ln}\left(\text{cSBP}/\text{cDBP}\right)}{\left(\text{SD}-\text{DD}\right)/\text{DD}}, \end{array}$$

where cSBP, cDBP, SD, and DD are central systolic and diastolic blood pressure and carotid systolic and diastolic diameter, respectively (Figure 1). *E*_*P*_ measures the ability of the arteries to change their dimensions in response to the pulse pressure caused by cardiac pulsatile ejection (pressure change required for (theoretic) 100% increase in diameter), whereas *β* is considered to be relatively independent of blood pressure levels [15].

### 2.3. Vascular Reactivity and Endothelial Function

Once baseline noninvasive arterial evaluation was carried out, we utilized the theoretical basis, general protocol, and methodological aspects of the VRT recommended by the guidelines for the ultrasound assessment of endothelial-dependent flow-mediated vasodilation of the brachial artery [7, 18]. For this purpose, participants were submitted to five minutes of ischemia by occluding left radial and cubital arteries using a pneumatic cuff placed around the left forearm (just below the elbow to at least 50 mmHg above pSBP) and several parameters of vascular reactivity were measured before, during, and after ischemia (Figure 1). The parameters used for the evaluation of EF are listed below.

#### 2.3.1. FMD, L-FMC, Brachial Biomechanics, and Shear Rate

Taking into account “gold standard” accepted methodology for the evaluation of EF (“recruitability”) and simultaneously for PWVcr measurement (see later), left brachial artery was visualized longitudinally above the antecubital crease using same high-resolution B-Mode ultrasound device mentioned earlier (Sonosite; MicroMaxx; USA) (Figure 1). Similarly, video sequences were recorded at rest, during forearm occlusion and after cuff deflation. Subsequently and similarly to the processing of carotid images, recordings were analyzed off-line using same automated step-by-step algorithm applied to each digitalized image that allows the brachial diameter waveform obtainment and FMD and L-FMC calculation [19]. Brachial local stiffness (*E*_*P*_ and *β*) was also determined by relating brachial arterial pressure and brachial diameters, as was explained earlier for carotid measurements.

FMD was quantified as the percentage of change in brachial DD, considering the basal levels and those measured one minute after cuff deflation:

(2) $$\begin{array}{c} \text{FMD}\%=\frac{{\text{DD}}_{\text{after cuff deflation}}-{\text{DD}}_{\text{baseline}}}{{\text{DD}}_{\text{baseline}}}\times 100. \end{array}$$

In addition, Doppler signals were performed to acquire blood flow velocity in baseline conditions and at specific moments during the reactive hyperemia period. Doppler signals were used to obtain the brachial* shear rate* (and its percentage of change), relating mean blood flow velocity (Vm (cm/s)) to brachial mean diameter (Dm) according to the following equations:

(3) $$\begin{array}{c} \text{SR}=\frac{\text{Vm}}{\text{Dm}},\\ \text{SR}\%=\frac{{\text{SR}}_{\text{ after cuff deflation}}-{\text{SR }}_{\text{baseline}}}{{\text{SR }}_{\text{baseline}}}\times 100. \end{array}$$

**Figure 2:**
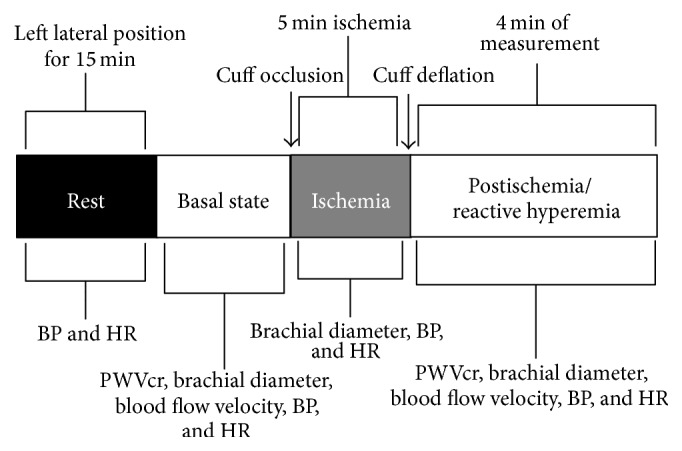
Representative diagram of the study protocol applied to evaluate changes in arterial parameters. PWVcr: carotid-to-radial pulse wave velocity; BP: blood pressure; HR: heart rate.

SR is an estimate of* shear stress* without accounting for blood viscosity [20] and was obtained for the characterization of the endothelial stimulus. The study protocol is represented in Figure 2.

#### 2.3.2. Carotid-to-Radial Pulse Wave Velocity

Noninvasive, carotid, and radial pressure waveforms were simultaneously obtained using strain gauge mechanotransducers (Motorola MPX 2050, Motorola Inc., Corporate 1303 E. Algonquin Road, Schaumburg, Illinois 60196, USA) by placing them on the skin over the carotid and radial sites (left hemibody). PWVcr was determined taking into account the given distance between these arterial sites (C-R Δ*x*) and the time delay (Δ*t*) between the carotid and radial waveforms onset (Figure 1). The algorithm used for the detection of the foot waves was described and explained in previous work [8]. Although a four-minute recording after cuff release was obtained, one minute after ischemia was the specific moment where the analysis was especially taken, according to previous reports [8, 16] (Figure 2). The PWVcr accepted variation coefficient was less than 7%.

PWVcr levels corresponding to baseline and to postischemia period were determined by averaging eight consecutive beats. After that, percent of change of PWVcr (with respect to basal levels) was quantified as follows:

(4) $$\begin{array}{c} \Delta \text{PWVcr}\%=\frac{{\text{PWVcr}}_{\text{after cuff deflation}}-{\text{PWVcr}}_{\text{baseline}}}{{\text{PWVcr}}_{\text{baseline}}}\times 100. \end{array}$$

All structural and function arterial evaluations were done by the same trained operator.

### 2.4. Statistics

The statistical analyses were performed using the Statistical Package for Social Sciences (version 22.0). Normality of the distribution of the data was examined using the Shapiro-Wilk test and *Q*-*Q* plot. All studied variables followed a normal distribution. All data are presented as mean value (MV) ± standard deviation (SD). Two-way analysis of variance (ANOVA) was employed for the evaluation of differences in variables within and between hypertensive and control pregnant women. Post hoc comparisons were done with the Bonferroni test. Differences in percentage of change of variables determined before and after the VRT (arterial diameter, PWV, and shear rate) were evaluated using two-tailed paired Student's *t*-test. Linear regression analyses were used to assess relationship between variables. *P* < 0.05 indicates significant statistical differences.

## 3. Results

Recordings were successfully obtained from all women and all studies were included in the analysis. The mean duration of the studies was 1 hour approximately and they were all well tolerated (without symptoms and/or complications).

The mean gestational age at examination of all the pregnant women was 35 ± 3 weeks. Demographic and anthropometric features and laboratory samples are shown in Table 1. Significant proteinuria in the daily urine collection could divide the group of hypertensive pregnant women in those with preeclampsia (with significant proteinuria >300 mg/24 hours, PE) and those with gestational hypertension (without or with only traces of proteinuria, GH). No significant proteinuria was found in HP. Maternal age, gestational age, and number of previous gestations were similar between study groups. Body weight and body mass index (BMI) were significantly higher in PE compared with HP and GH (*P* < 0.05). Uric acid levels were within normal values in HP and GH, while in PE they were abnormally increased [21].

Baseline cardiovascular characteristics are given and compared in Table 2. Baseline peripheral SBP, DBP, and MAP levels were significantly higher in PE and GH in comparison with HP (*P* < 0.001). No peripheral BP differences were found among groups with hypertension (GH versus PE). However, differences were found in these groups when central hemodynamics is analyzed. For example, cPP was different between study groups. In addition, PE showed higher values of cSBP compared with GH (*P* = 0.004), without differences in cPP and cDBP. When compared with HP women, levels of cSBP and cDBP in women with PE and GH were higher.

AIx@75 and the amplification ratio, two composite measures of systemic arterial stiffness and wave reflection amplitude, were analyzed and are presented in Table 2. AIx@75 was significantly higher in PE with respect to GH and HP (24.3 ± 5.7% versus 11.8 ± 7.6 and 12.2 ± 12.4%, resp.; *P* = 0.05). No significant differences were found in this parameter between GH and HP. On the other hand, amplification ratio (cPP/pPP) was only statistically different between PE and GH, with PE having the lowest values.

When analyzing muscular peripheral arteries (i.e., brachial artery) by local (*E*_*P*_ and *β*) and regional arterial stiffness parameters (PWVcr), no differences were found among groups. However, CCA and aorta (i.e., elastic arteries) showed meaningful differences in stiffness. In general, PE showed stiffer elastic arteries with respect to other groups. For example, right CCA *E*_*P*_ was significantly increased (duplicating approximately its values) in PE with respect to HP and GH (*P* < 0.001 and *P* = 0.004, resp.). Similar tendencies were noticed in *β* from the right CCA but not reaching statistical differences, indicating that changes in carotid artery stiffness in PE and GH are pressure-dependent. On the left side, differences were observed in *E*_*P*_ comparing PE and HP, and similar tendencies were maintained for *β*.

Finally, hypertensive pregnant women showed higher values of PWVcf (regional aortic stiffness) compared with HP women. However, no differences were found between the groups with hypertension, although women with PE had a tendency to show higher values (*P* = 0.14).

None of the groups (HP, GH, or PE) presented atherosclerotic plaques. However, structural differences were noticed on the right common carotid artery. Right, but not left, CIMT was significantly elevated in PE with respect to HP women (*P* = 0.010).

Taking into account the VRT (vascular reactivity test), all groups evoked endothelial stimulus (reactive hyperemia) evaluated by changes in shear rate before and after cuff deflation (*P* < 0.001). In addition, peak SR and ΔSR% were the same among groups (*P* = 0.86 and *P* = 0.39, resp.) (Table 3). No significant changes were found in heart rate or blood pressure intra- and intergroup before and after cuff deflation, ensuring stable hemodynamic conditions during the maneuver (data not shown).

Regarding the FMD, all of them showed a dilatation of the brachial artery with respect to the basal state but without statistical significance in women with PE. As was expected, HP women showed quantitatively the highest FMD response (9.4 ± 3.0%; *P* < 0.001), while women with GH and PE reached the lowest values (3.6 ± 3.3%; *P* = 0.021; 2.2 ± 2.9%; *P* = 0.081, resp.). FMD mean values of GH and PE compared to HP were significantly different (*P* < 0.001). As was mentioned above, baseline PWVcr were similar among groups. One minute after the cuff deflation, PWVcr decreased only in HP (7.0 ± 1.6 to 5.9 ± 0.8 m/s, *P* < 0.01). However, GH showed a blunted hyperemic PWVcr response (7.1 ± 0.9 to 7.0 ± 0.8 m/s; *P* = 0.627), while PE showed a tendency to increase arterial stiffness (6.0 ± 1.1 to 6.4 ± 1.3 m/s; *P* = 0.06). PWVcr percentage changes [ΔPWVcr (%)] differed comparing HP women with women with GH (−13.9% versus −0.9%; *P* < 0.01) and with PE (−13.9% versus +7.0%; *P* < 0.01). No differences were found between GH and PE (*P* = 0.221).

L-FMC of the brachial artery was different according to the pregnancy status (*P* < 0.001). Maximal vasoconstriction (negative values) was observed in HP women (−7.8 ± 3.7%, *P* < 0.001) followed by women with GH (−4.5 ± 2.1%, *P* < 0.001), while women with PE did not reach significant arterial constriction during the cuff inflation (−0.7 ± 3.5; *P* = 0.576) (Table 3). There were no differences in L-FMC between PE and GH.

**Figure 3:**
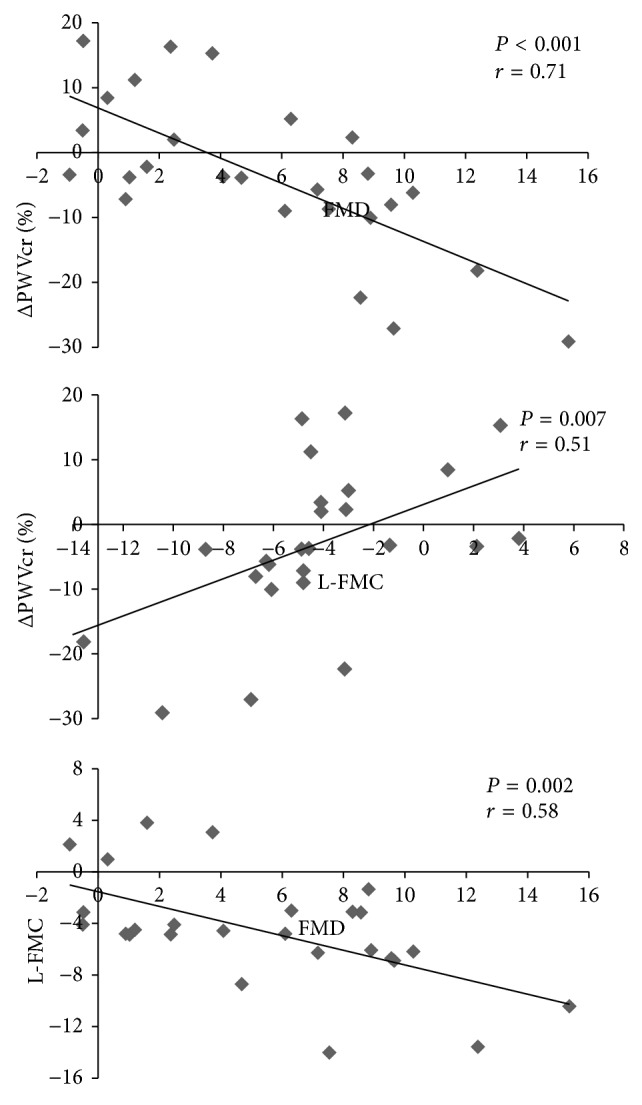
Correlation analysis between EF parameters. FMD: flow-mediated dilation; L-FMC: low-flow-mediated constriction; ΔPWVcr%: percentage of change in carotid-to-radial pulse wave velocity.

Demographic, anthropometric, and laboratory variables shown in Table 1 did not significantly correlate with any of the arterial parameters. In addition, there was no significant correlation between parameters of EF (i.e., FMD, L-FMC, and ΔPWVcr%) and AIx@75 or amplification ratio (data not shown). However, a low but statistically significant correlation was found between baseline PWVcf and L-FMC (*r* = 0.45, *P* = 0.04), without reaching statistical significance with other EF parameters. A significant correlation between FMD, L-FMC, and ΔPWVcr% was seen among these parameters in the whole study population (Figure 3).

## 4. Discussion

The present study is, to our knowledge, the first one to determine and assess simultaneously, in a group of healthy and hypertensive pregnant women, the vascular reactivity or EF by using three different but complementary methods in conjunction with the determination of central and peripheral arterial structural and functional parameters.

The main results of this work were as follows: (1) central aortic blood pressure and wave reflections as well as elastic (aortic and carotid) arteries stiffness are increased in PE, with respect to peripheral blood pressure-matched GH and HP, and (2) PE showed both resting (L-FMC) and recruitable (FMD and ΔPWVcr%) endothelial dysfunction.

Among the methods that allow measurement of vascular reactivity or EF in the clinical setting, FMD has rapidly gained popularity because of its simplicity, reproducibility, and noninvasiveness [7, 18]. However, as was mentioned earlier, one important limitation of FMD is that it only provides information about the “recruitability” of EF (i.e., its responsiveness to a specific stimulus) and not about concerning “resting” EF (i.e., release of endothelial autacoids before FMD measures are initiated) [11]. We here analyze in hypertensive pregnant women both types of functional aspects of EF: “endothelial recruitability” through FMD and PWVcr changes and “resting endothelial tone” through L-FMC. The magnitude of FMD observed in HP in response to VRT was similar to that described in previous reports [9, 10]. As it was expected, hypertensive pregnant women showed a reduction in FMD with respect to HP, in coherence with greater degrees of endothelial dysfunction [22, 23]. It is noteworthy that only PE did not reach statistical significance in the dilation of the brachial artery, obtaining a more complete blunted response. Although the FMD of PE was numerically lower than those from GH, this difference did not reach statistical significance. This could be attributed or not attributed to the magnitude of the standards deviation of the mean due to the low sample size. Nevertheless, approximately 25% of women initially diagnosed with GH will develop PE [24]. Therefore, the vascular profile from pregnant women with GH who might develop PE could be quite similar to those women with PE. However, there is a lack of information that compares FMD between groups with PE and GH and only few studies directly analyze this issue. According to Quinton et al., the FMD at one minute of the cuff deflation was not different between the GH and PE in women who were not receiving any medication, while there were statistical differences between these groups when women were receiving medical treatment [25]. Nevertheless, in a prospective study conducted by Filho et al., they did not find differences in FMD of the brachial artery in patients with two different forms of hypertensive disorders of pregnancy [26].

When analyzing changes in arterial stiffness due to VRT, HP showed the major reduction in PWVcr values. On the other hand, women with hypertension showed not only a blunted response in PWVcr changes but also, in PE, a tendency to increase arterial stiffness one minute after the cuff deflation was evidenced. Indeed, by means of this method, changes of PWVcr in PE tended to be higher in comparison to GH, indicating probably greater degree of impairment of EF. It is noteworthy that all participants showed the same increase in blood flow velocity with respect to basal conditions after cuff deflation (“endothelial stimulus”), and variables such as baseline levels of PWVcr, basal brachial diameter, blood pressure, and gestational age were similar among the groups.

Taking into account “resting” endothelial tone, our results show that, during cuff inflation, brachial artery responses varied between the studied groups. L-FMC of the brachial artery was significant only in HP and GH, without any constriction in PE, suggesting that PE develop also basal endothelial dysfunction. Although L-FMC was firstly described and assessed at the radial artery, Spiro et al. evidenced later that this phenomenon also occurs in healthy subjects at the brachial artery and it can be measured reliably [27]. Studies agree that radial artery vasoconstriction occurs during cuff inflation in nonpregnant women [12, 28, 29], whereas recent studies examining the brachial diameter during occlusion demonstrate conflicting results [27, 28, 30–32]. Weissgerber et al. did not evidence L-FMC in the brachial artery in pregnant women [29]. Differences in cardiovascular profile, methodological issues, and interobserver variability could explain the widely variable results. For example, we here measure L-FMC of the brachial artery in a regimen of low but not zero blood flow (as it occurs in the radial artery) in a level that is upstream of the occlusion site. Therefore, the magnitude of reduced blood flow in the brachial artery and its relationship with the basal levels (endothelial “negative” stimulus for vasoconstriction) should surely yield different brachial responses.

As it was previously reported, we found that women with PE showed marked structural and functional alterations in peripheral and central hemodynamics [4, 33–35]. PE had a strong tendency to present higher values in practically all studied parameters related to central hemodynamics. For instance, central SBP, AIx@75, CCA *E*_*P*_, and PWVcf were significantly higher in PE with respect to HP. We also found differences in central hemodynamics between women with hypertension, but this was not the rule as it was for PE versus HP. Only cSBP, AIx@75, and right CCA *E*_*P*_ were markedly augmented in comparison to GH. These findings were not due to differences in peripheral blood pressure, which was elevated to a similar degree in both types of hypertensive states. These pieces of information analyzed together indicate that women with hypertensive disorders in pregnancy (mainly PE) have increased central BP overload, central arterial stiffness, and amount of wave reflections, probably related to a vasoconstriction state due to endothelial dysfunction [35]. Altered central hemodynamics in PE may signify an inadequately increased left ventricle afterload and myocardial oxygen demand in the mother circulation, as well as hemodynamic disturbances transmitted to the fetal circulation [36].

Blunted FMD, L-FMC, and PWVcr changes evidenced in PE are in consonance with the plasma uric acid levels that were found elevated only in this group. In previous reports, hyperuricemia was associated with an increase of plasma xanthine oxidase activity and/or a reduction in antioxidant systems [37] related to increased formation of reactive oxygen species and endothelial dysfunction [38]. Differences in BMI were found among the groups. Increased BMI in PE could reflect an associated overweight/obesity state, differences in Na^+^ and body fluid retention by the hemodynamic overload due to the hypertensive condition, or a combination of both.

We found a significant correlation between the EF parameters. Our results indicate that brachial artery responses to inflation and deflation of the cuff related to endothelial dynamics could share some vascular mechanism. However, there are confusing results around the FMD and L-FMC correlation, with variable results depending on the analyzed artery (brachial versus radial) and type of physiological or pathophysiological circumstance [11–13, 27]. This emphasizes again the complexity of the study of “endothelial functions.” Although both L-FMC and FMD are an expression of the vascular reactivity in response to changes in blood flow, their relationship is neither conceptually simple nor mathematically linear [13]. On the other hand, when analyzing the relationship between FMD and PWVcr the analysis can also be a little more complex. According to Moens and Korteweg equation, PWV is determined by arterial diameter and also by the elastic modulus [5]. If post-VRT changes in PWVcr in PE and GH would have followed only the changes in brachial diameter (FMD), the obtained changes in PWVcr would have shown an equal behavior to the geometrical change (change in diameter) [39]. However, in accordance with the obtained values in the groups with hypertension, a dissociation among these variables was evidenced, with an increase of arterial diameter (which would reduce the levels of PWVcr) without significant changes in PWVcr levels (or even a trend to increase in PE) after the cuff deflation period. This discordance between parameters behavior in response to the VRT indicates an increase in the elastic modulus in parallel with changes in the arterial diameter. Thus, at least in PE, we evidenced a reciprocal and simultaneous change in the vascular wall intrinsic properties and the brachial diameter.

An impaired response to changes in blood flow in a concrete vascular ledge (e.g., brachial artery), without simultaneous adequate change both in brachial diameter and in arterial stiffness, could have important hemodynamic consequences. At first, a reduction in the vasodilator reserve related to endothelial dysfunction as it was seen in other pathophysiological circumstances [40] could implicate an incapacity of the arterial system to determine an appropriate vascular adjustment against hemodynamic changes in the long (fetal growth) and even in the short term (exercise, change of position, etc.). Second, an impaired capability of response to hemodynamic changes due to endothelial dysfunction could yield other functional cardiovascular alterations that was seen in PE, like increased left ventricle afterload and diastolic dysfunction [41]. This point is in consonance with altered values of central parameters found in PE mentioned above. The important additional information brought by introduction changes in PWVcr and L-FMC, together with the information of central and peripheral hemodynamics, is that these variables provide information concerning a different aspect of vascular reactivity and EF, therefore complementing (and not overlapping with) the information provided by FMD. This vascular approach may provide a more comprehensive assessment of vascular state and endothelial function in hypertensive disease of pregnancy.

The sample size of our study was relatively small. However, our findings were statistically significant and, by definition, this indicates that the study was adequately statistically powered. Our technical approaches including the use of both multiple automated and semiautomated edge-detection/point software in ultrasound image and pressure wave assessment are largely operator-independent and also empower our findings [28]. Given the means of the different variables and SDs observed in previous works and in the present sample, twenty-five subjects (*n* = 25) of the total sample size (the sum of the sizes of comparison groups) would be required to detect a statistically significant effect of the pregnancy status with at least 80% of power [42].

The detection of pregnant women who finally will develop PE remains a clinical challenge. There is no isolated technique which satisfies completely this purpose with enough accuracy. At the present time, different combinations of clinical risk factors, biochemical markers, and Doppler ultrasound of the uterine arteries are recommended [43]. The detection rate of PE using only one clinical model of screening that includes risk factors (e.g., nulliparity, maternal age, family history of PE, etc.) is 45.3%, while only with Doppler ultrasound of uterine arteries at the second trimester it is 63.1% and with a combined approach it reaches 67.5%, with a 25% of false positive rate [5]. The clinical importance of improving detection of PE can also be stressed when confidential enquiries are analyzed, showing that in a substantial proportion of cases of fetal death due to preeclampsia a different management might have altered the outcome [44]. Moreover, the evidence demonstrates that administration of antiplatelet agents (primarily low dose of aspirin in different trials) to well-selected women leads to a significant reduction in the risk of developing preeclampsia and its serious consequences [45]. For these reasons, an accurate prediction of preeclampsia or early diagnosis may, therefore, allow more efficient allocation of resources for monitoring and improving maternal and perinatal outcomes [1, 2]. On the other hand, the extensive and growing information that links endothelial dysfunction/arterial damage with pathophysiology of PE motivates researchers and clinicians to evaluate arterial parameters (including endothelial function) in this clinical setting. Additionally, there is a need to count with a more comprehensive assessing EF in a patient in concrete. In that sense, the inclusion of validated arterial parameters and a more complete EF evaluation in the contemporary assessment of preeclampsia into multiparametric models could improve prediction of PE [43]. In this small study, which addresses the feasibility of measuring these parameters simultaneously, simply, and noninvasively, we found encouraging results that we believe warrant further investigation in order to contribute to the early recognition of preeclampsia.

## 5. Conclusion

This is the first study that measures and analyzes, in the same pregnant women, central and peripheral hemodynamics and EF by using different parameters that offer additional and complementary information. “Resting and recruitable” EF from pregnant women can be assessed by using PWVcr changes and L-FMC, respectively. Impaired EF which follows hypertensive disorder of pregnancy, mainly PE, showed both “resting and recruitable” endothelial dysfunctions.

Central aortic pressure and wave reflections as well as stiffness of elastic arteries are improperly increased in PE. Future studies will have to determine if incorporation of these pieces of information together, assessing basal state and functional reserve or capability of response of the vascular system into multiparametric models that include clinical, obstetric, and laboratory variables and Doppler ultrasound of uterine arteries, will be able to improve contemporary prediction of preeclampsia (from healthy pregnancy and from gestational hypertension). Hopefully, this could change the clinical management and prognosis of the pregnant women with PE. The clinical impact of these results remains uncertain but merits further investigation.

## Figures and Tables

**Table 1:** Demographic and laboratory samples characteristics of the study populations according to pregnancy status.

	Healthy pregnant (HP)	Gestational hypertension (GH)	Preeclampsia (PE)	*P* value^*∗*^
	MV ± SD	MV ± SD	MV ± SD	
*n*	10	8	8	
Age (years)	29.4 ± 6.2	30.9 ± 6.7	33.6 ± 5.9	0.373
Gestational age (weeks)	34.6 ± 3.7	34.9 ± 2.9	36.3 ± 2.2	0.502
Number of gestations (*n*)	1.8 ± 2.1	4.5 ± 3.8	3.5 ± 4.5	0.286
Weight (kg)	67.1 ± 7.3	76.5 ± 10.9	97.0 ± 12.6^a,b^	<0.001
Height (cm)	157.6 ± 6.8	159.6 ± 5.3	160.3 ± 8.6	0.703
BMI (kg/m^2^)	27.1 ± 3.6	30.2 ± 5.4	38.1 ± 6.6^a,b^	0.001
Creatinine (mg/dL)	0.7 ± 0.1	0.6 ± 0.1	0.6 ± 0.1	0.664
Uric acid (mg/dL)	3.2 ± 1.1	4.1 ± 1.4	6.7 ± 2.5^a,b^	0.001
24-hour proteinuria (g)	0.0 ± 0.1	0.2 ± 0.1^a^	0.5 ± 0.1^a,b^	<0.001
SGOT (UI/L)	12.2 ± 3.2	15.7 ± 5.9	14.5 ± 2.5	0.722
SGPT (UI/L)	11.5 ± 5.2	13.7 ± 6.0	11.8 ± 4.1	0.587
LDH (UI/L)	320.5 ± 59.7	347.8 ± 49.8	386.9 ± 93.3	0.846
Hematocrit (%)	31.7 ± 3.7	34.2 ± 2.9	33.7 ± 2.0	0.681
Platelets (*μ*L^−1^)	213.0 ± 78.0	195.4 ± 60.0	202.6 ± 45.6	0.862

Values are expressed as mean value (MV) ± standard deviation (SD). Post hoc test with multiple comparisons: a and b indicate *P* < 0.05 with respect to HP and GH, respectively. All comparisons were determined using ANOVA + Bonferroni test. HP: healthy pregnancies; GH: gestational hypertension; PE: preeclamptic pregnancies; BMI: body mass index; SGOT: serum glutamic-oxaloacetic transaminase; SGPT: serum glutamic-pyruvic transaminase; LDH: lactate dehydrogenase. ^*∗*^Between groups.

**Table 2:** Baseline cardiovascular characteristics of the study populations according to pregnancy status.

	Healthy pregnant (HP)	Gestational hypertension (GH)	Preeclampsia (PE)	*P* value^*∗*^
	MV ± SD	MV ± SD	MV ± SD	
Heart rate (bpm)	81.8 ± 15.9	84.8 ± 9.7	85.3 ± 14.0	0.844
Peripheral SBP (mmHg)	111.8 ± 8.2	139.4 ± 6.0^a^	145.5 ± 7.5^a^	<0.001
Peripheral DBP (mmHg)	63.5 ± 9.5	78.9 ± 11.9^a^	84.6 ± 8.8^a^	<0.001
MBP (mmHg)	79.6 ± 5.8	99.0 ± 6.3^a^	105.9 ± 7.3^a^	<0.001
Peripheral PP (mmHg)	50.7 ± 18.6	58.6 ± 11.4	58.0 ± 12.7	0.571
Central SBP (mmHg)	96.7 ± 6.7	118.9 ± 3.9^a^	130.3 ± 5.8^a,b^	<0.001
Central DBP (mmHg)	63.5 ± 9.5	80.4 ± 11.2^a^	85.6 ± 10.0^a^	<0.001
Central PP (mmHg)	33.2 ± 11.4	35.5 ± 7.1	42.8 ± 10.8	0.041
Amplification ratio	1.5 ± 0.2	1.7 ± 0.2	1.4 ± 0.2^b^	<0.001
AIx@75 (%)	12.2 ± 12.4	11.8 ± 7.6	24.3 ± 5.7^a,b^	0.018
Carotid-to-femoral PWV (m/s)	5.6 ± 0.8	7.1 ± 0.8^a^	8.2 ± 1.2^a^	<0.001

Carotid-to-radial PWV (m/s)	7.0 ± 1.6	7.1 ± 0.9	6.0 ± 1.1	0.159
Brachial SR (s^−1^)	117.9 ± 43.1	102.8 ± 26.7	93.4 ± 43.5	0.417
Brachial SD (mm)	3.8 ± 0.3	3.9 ± 0.4	4.3 ± 0.5	0.149
Brachial DD (mm)	3.7 ± 0.3	3.7 ± 0.4	4.1 ± 0.4	0.210
Brachial *E*_*P*_ (mmHg)	1010 ± 464	1188 ± 832	1183 ± 350	0.754
Brachial *β*	11.4 ± 5.0	11.1 ± 8.3	11.3 ± 3.4	0.953

Right CCA SD (mm)	7.1 ± 0.6	7.1 ± 0.6	7.1 ± 0.5	0.988
Right CCA DD (mm)	6.5 ± 0.6	6.5 ± 0.6	6.7 ± 0.5	0.749
Right *E*_*P*_ (mmHg)	355 ± 102	434 ± 161	709 ± 185^a,b^	<0.001
Right CCA *β*	4.56 ± 1.47	5.24 ± 3.07	6.95 ± 1.58	0.073
Right CIMT (mm)	0.47 ± 0.10	0.55 ± 0.08	0.66 ± 0.21^a^	0.036

Left CCA SD (mm)	7.1 ± 0.4	7.2 ± 0.3	7.1 ± 0.7	0.932
Left CCA DD (mm)	6.5 ± 0.4	6.6 ± 0.2	6.7 ± 0.6	0.637
Left CCA *E*_*P*_ (mmHg)	376 ± 153	471 ± 182	714 ± 256^a^	0.005
Left CCA *β*	4.8 ± 2.0	5.6 ± 3.1	7.0 ± 2.4	0.205
Left CIMT (mm)	0.51 ± 0.09	0.55 ± 0.08	0.59 ± 0.10	0.162

Values are expressed as mean value (MV) ± standard deviation (SD). Post hoc test with multiple comparisons: a and b indicate *P* < 0.05 with respect to HP and GH, respectively. All comparisons were determined using ANOVA + Bonferroni test. HP: healthy pregnancies; GH: gestational hypertension; PE: preeclamptic pregnancies; SBP: systolic blood pressure; DBP: diastolic blood pressure; MBP: mean blood pressure; PP: pulse pressure; AIx@75: augmentation index adjusted to a heart rate of 75 bpm; PWV: pulse wave velocity; SR: shear rate; SD and DD: systolic and diastolic diameter, respectively; *E*_*P*_: Peterson's elastic modulus; *β*: stiffness index; CCA: common carotid artery; CIMT: carotid intima-media thickness. ^*∗*^Between groups.

**Table 3:** VRT-related changes in vascular parameters of the study groups according to pregnancy status.

	Healthy pregnant (HP)	Gestational hypertension (GH)	Preeclampsia (PE)	*P* value^*∗*^
	MV ± SD	MV ± SD	MV ± SD	
FMD (%)	9.4 ± 3.0	3.6 ± 3.3^a^	2.2 ± 2.9^a^	<0.001
L-FMC (%)	−7.8 ± 3.7	−4.5 ± 2.1	−0.7 ± 3.5^a^	<0.001
ΔPWVcr (%)	−13.9 ± 9.4	−0.9 ± 6.9^a^	7.0 ± 8.5^a^	<0.001
Peak shear rate (s^−1^)	231.7 ± 72.6	237.6 ± 67.5	219.5 ± 59.5	0.86
ΔShear rate (%)	113.8 ± 87.4	131.2 ± 53.7	168.6 ± 97.1	0.39

Values are expressed as mean value (MV) ± standard deviation (SD). Post hoc test with multiple comparisons: a indicate *P* < 0.05 with respect to HP and GH, respectively. All comparisons were determined using ANOVA + Bonferroni test. HP: healthy pregnancies; GH: gestational hypertension; PE: preeclamptic pregnancies; FMD: flow-mediated dilation; L-FMC: low-flow-mediated constriction; PWVcr: carotid-to-radial pulse wave velocity. ^*∗*^Between groups.
